# Supplementary material for: Galanin neurons in the ventrolateral preoptic area promote sleep and heat loss in mice
Source: Nat Commun. 2018 Oct 8;9:4129. doi: 10.1038/s41467-018-06590-7 (PMC6175893; doi:10.1038/s41467-018-06590-7)
Supplement: Supplementary file 1 — Supplementary Information [file 41467_2018_6590_MOESM1_ESM.pdf]

Supplementary information for:

Galanin neurons in the ventrolateral preoptic area promote sleep and heat loss in mice

Kroeger et al.

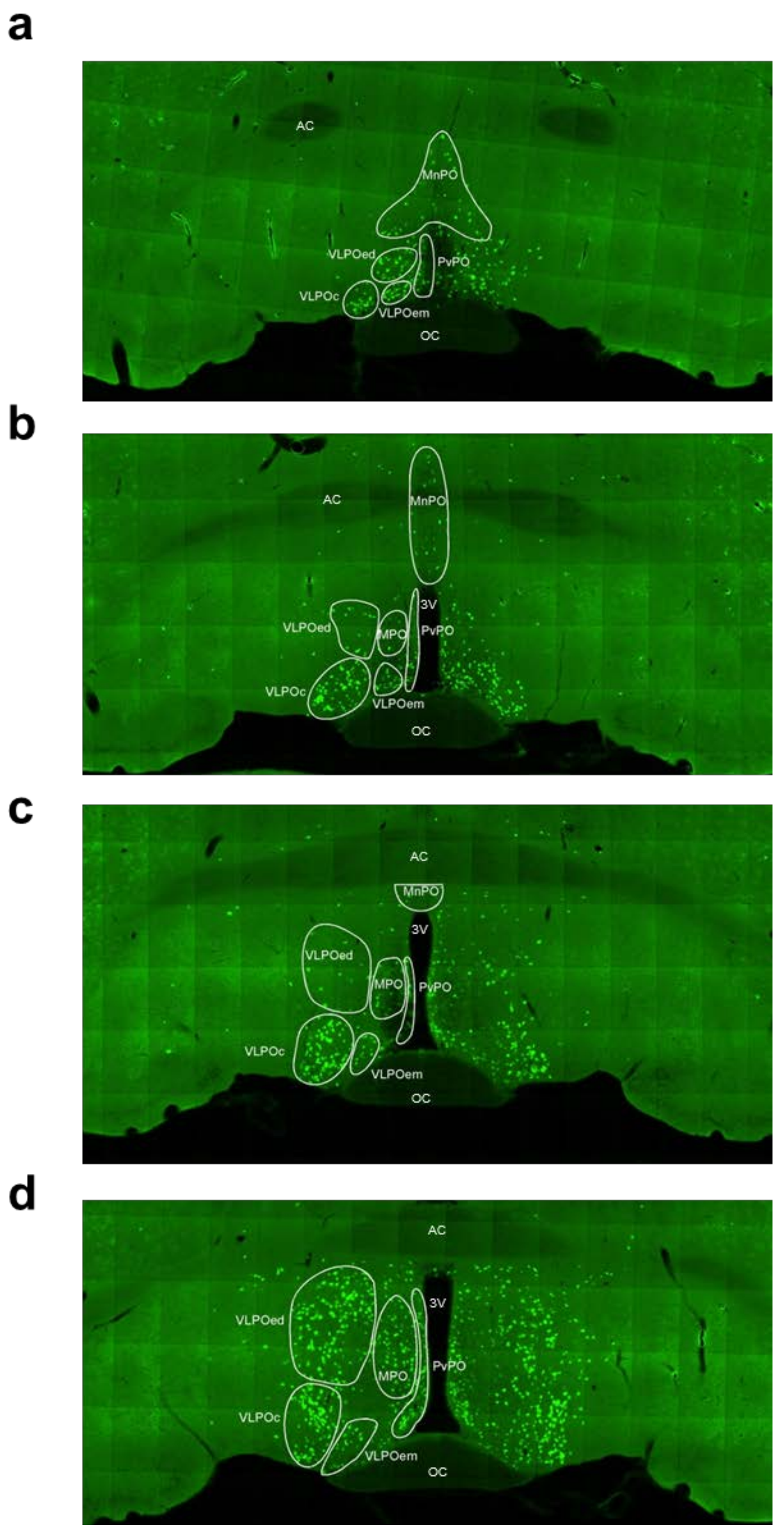

Supplementary Figure 1. Distribution of GAL neurons in the VLPO in GAL-Cre-L10 GFP reporter mice. (**a-d**) Fluorescent images of 40 μm sections at the levels of VLPO corresponding to approximately AP: +0.38, +0.26, +0.14, 0.0 from bregma in the mouse atlas of Frankin and Paxinos,[1] showing GAL neurons in green. The larger cells of the VLPO core (VLPOc), and its

medial (VLPOem) and dorsal (VLPOed) extensions, demarcate these structures, whereas the median (MnPO), medial (MPO), and periventricular preoptic (PvPO) nuclei contain smaller neurons. Levels **b** and **c** were used as templates for plotting injection sites in Main Figures **1** and **4**. AC, Anterior commissure; OC, Optic chiasm; 3V, 3rd ventricle.

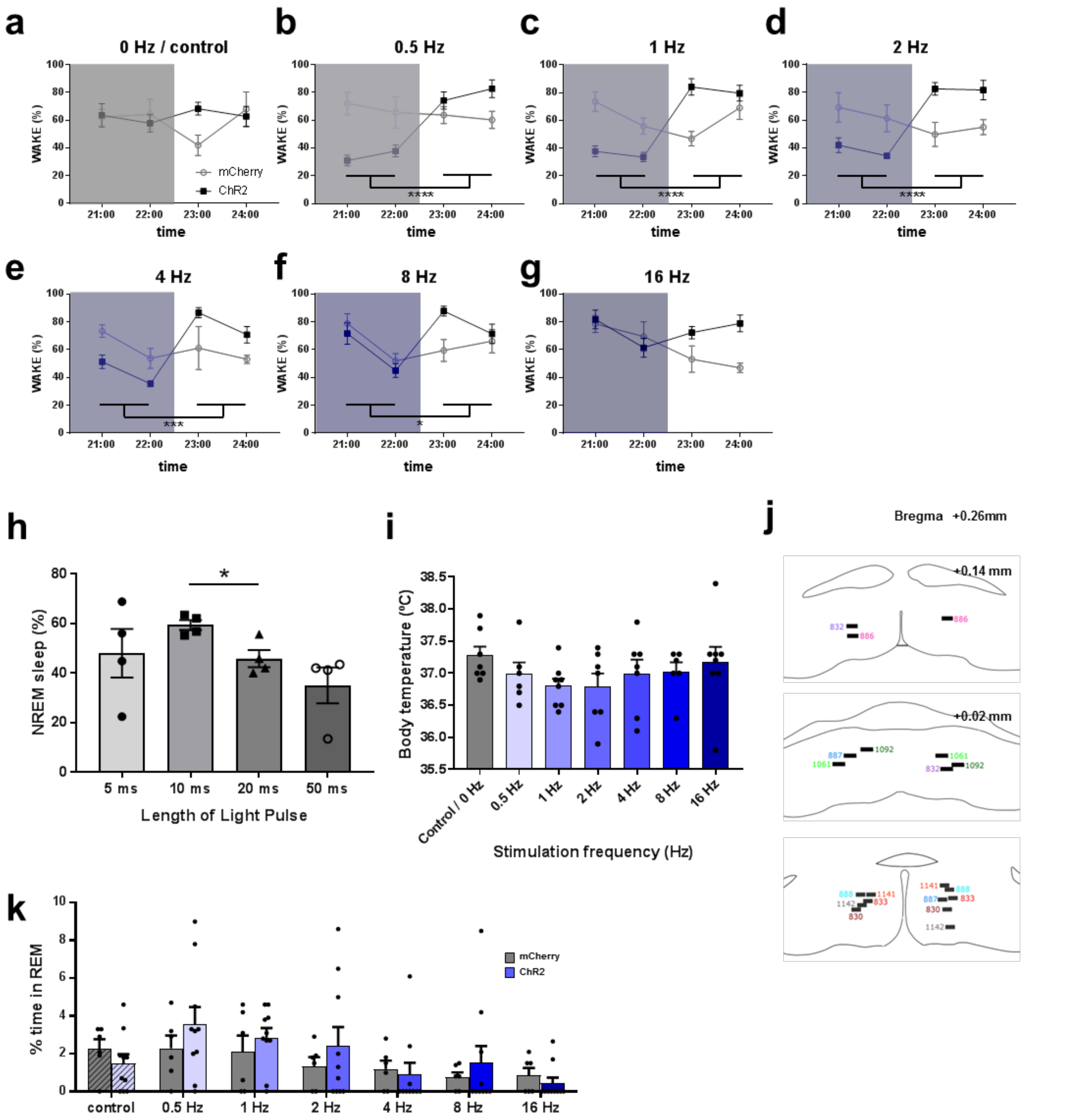

Supplementary Figure 2. Wake rebound after optogenetic activation of VLPO^{GAL} neurons. (**a-g**) Hourly percentages of wake during and 2 h immediately after photoactivation of VLPO^{GAL}-mCherry neurons (n = 6 mice) and VLPO^{GAL}-ChR2 neurons (n = 10 mice) at different stimulation frequencies. Continuous pulsed laser light for 2 h (21:00-23:00 h; colored areas) that produced an increase in sleep and reduction in wake was followed by a period of increased wake (‘wake rebound’) during the subsequent 2 h in VLPO^{GAL}-ChR2 mice at stimulation frequencies of 0.5-4 Hz; Paired t-tests. (**h**) Percent time in NREM sleep during photostimulation of VLPO^{GAL} neurons with different durations (5-50 ms) of 1 Hz laser light pulses for 2 h (21:00-23:00 h); one-way RM

ANOVA for treatment for $VLPO^{GAL}$-ChR2 mice was not significant (F(1.819, 5.457)=2.961, *P*=0.1360; n = 4 mice). (**i**) Average body temperature during the 2 h stimulation period (21:00-23:00 h) in $VLPO^{GAL}$-ChR2 mice (n = 10) at different stimulation frequencies. There were no overall significant differences when comparing all the groups using Kruskal-Wallis test (*P*=0.5136). (**j**) Location of optical fiber tips (n = 10 mice; numbers represent the animal IDs) in VLPO at three levels (AP: +0.26; AP: +0.14 and AP: + 0.02 as per mouse atlas of Paxinos and Franklin[76]). Note that the fiber tip locations in all cases were sufficiently lateral to avoid illuminating the medial, median, or periventricular preoptic nuclei. (**k**) Percent time spent in REM sleep during the 2 h photostimulation (from 21:00 to 23:00) of $VLPO^{GAL}$ neurons at various stimulation frequencies (control, 0.5 Hz, 1 Hz, 2 Hz, 4 Hz, 8 Hz and 16 Hz). We found no significant difference in percent time spent in REM sleep between ChR2-mice vs. mCherry-mice (two-way repeated measures (RM) ANOVA for 2 h of recording time for 'virus type' and 'stimulation frequency' followed by Sidak's *post hoc* test (virus type: $F(1,14) = 0.2331$, $p=0.6367$; stimulation frequency: $F(6,84)=4.076$, $p<0.0012$, n = 10 $VLPO^{GAL}$-ChR2 mice vs. n = 6 $VLPO^{GAL}$-mCherry mice). (Data are Mean ± SEM. *p < 0.05, ***p < 0.001, ****p < 0.00001.

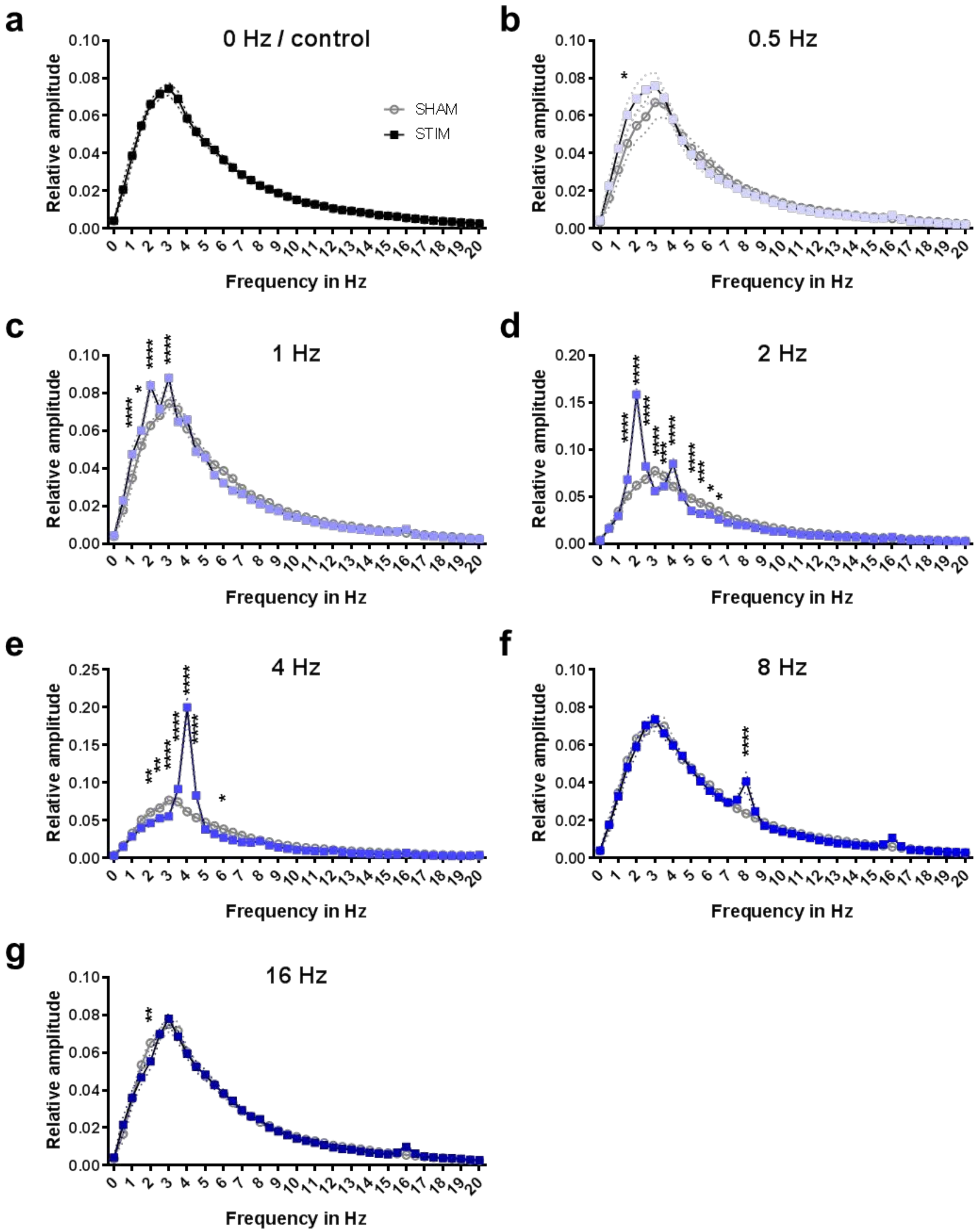

Supplementary Figure 3. EEG power spectra during optogenetic activation of VLPO^{GAL} neurons. (**a-g**) EEG power spectra during 0 - 16 Hz optical stimulation in VLPO^{GAL}-ChR2 mice. The EEG power spectrum for stimulation rates of 1, 2, 4, and 8 Hz showed clear peaks of amplitude at the frequency of the stimulus and its first harmonic suggesting that stimulation of

VLPO^{GAL} neurons at those frequencies entrained the cortical EEG. Even stimulation at 16 Hz caused a small peak of EEG power at 16 Hz. Only stimulation at 0.5 Hz did not cause such a peak, but it is possible that overlap of increased power at 0.5 Hz and its multiples may have blurred together, causing the shift of the peak to lower frequencies. Two-way RM ANOVAs for each stimulation frequency in the SHAM and STIM condition during NREM sleep in the 2 h period of treatment (21:00-23:00 h) for ‘EEG frequency’ and ‘optical treatment’ (sham stimulation vs. laser light stimulation), followed by Sidak’s *post hoc* test (0 Hz - control condition: no significant differences; 0.5 Hz condition: no significant differences; 1 Hz: EEG frequency $F(40,640)=389.8$, $p<0.0001$, optical treatment $F(1,16)=4.759$, $p=0.0444$; 2 Hz: EEG frequency $F(40,640)=404.5$, $p<0.0001$, optical treatment $F(1,16)=12.43$, $p=0.0028$; 4 Hz: EEG frequency $F(40,640)=237.7$, $p<0.0001$, optical treatment $F(1,16)=4.109$, $p=0.0597$; 8 Hz: $F(40,640)=293.3$, $p<0.0001$, optical treatment $F(1,16)=1.125$, $p=0.3045$;16 Hz: EEG frequency $F(40,640)=301$, $p<0.0001$, optical treatment $F(1,15)=7.087$, $p=0.0178$; n =10 mice). Data are Mean ± SEM. * P < 0.5, **p < 0.01, ***p < 0.001, ****p < 0.00001.

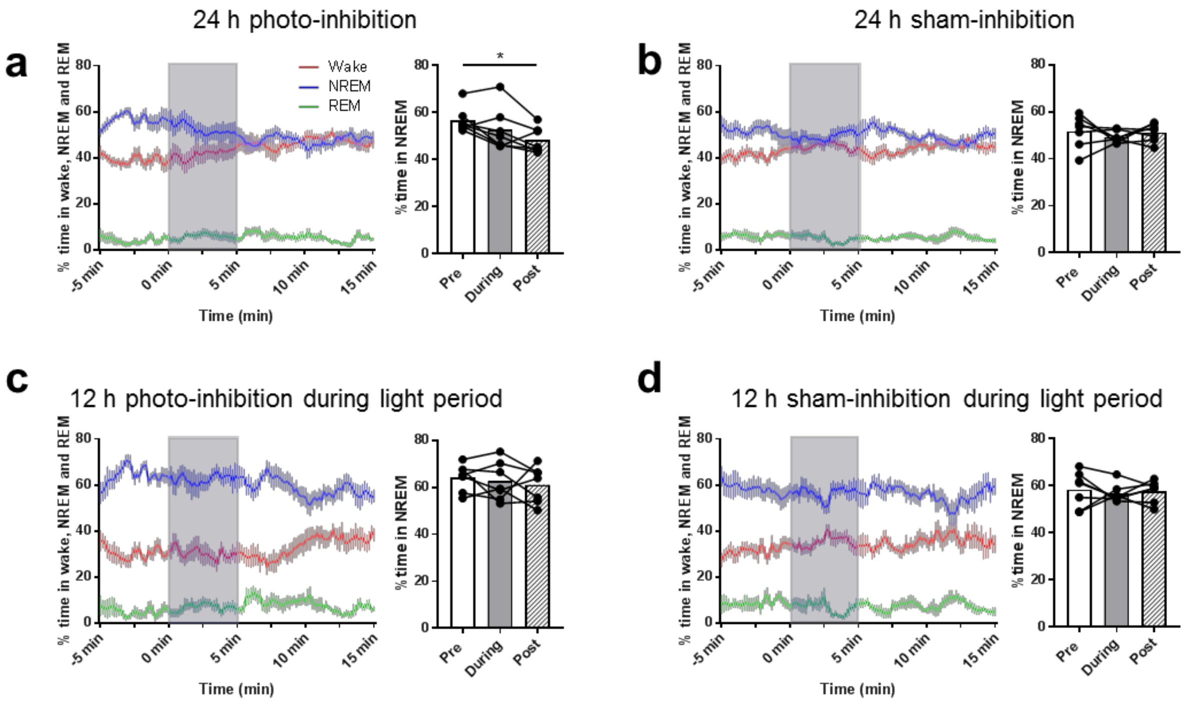

Supplementary Figure 4. Sleep-wake changes after photoinhibition of VLPO^{GAL} neurons. (**a**) Photoinhibition of VLPO^{GAL} neurons significantly decreased the percent time spent in NREM sleep over a 24 h period. We applied 5 min light pulses (593.5 nm; 10 mW/mm^2 at the fiber tip) every 30 mins for 24 h and compared 5 min periods 'before', 'during', and 'after' the photoinhibition using a one-way repeated measures ANOVA for 'treatment groups', followed by Tukey's *post-hoc* test ($F(2, 12)=7.252$, $p=0.0086$, n = 7 mice). We found a significant decrease in NREM sleep (**a**) between 'pre' and 'post' stimulation periods whereas sham inhibition did not alter NREM sleep (**b**). The decrease in NREM sleep after photoinhibition was more pronounced during the dark period (See main Figure 3d), but photoinhibition during the light period had no effect on NREM sleep (**c,d**). Data are Mean ± SEM. *p < 0.5.

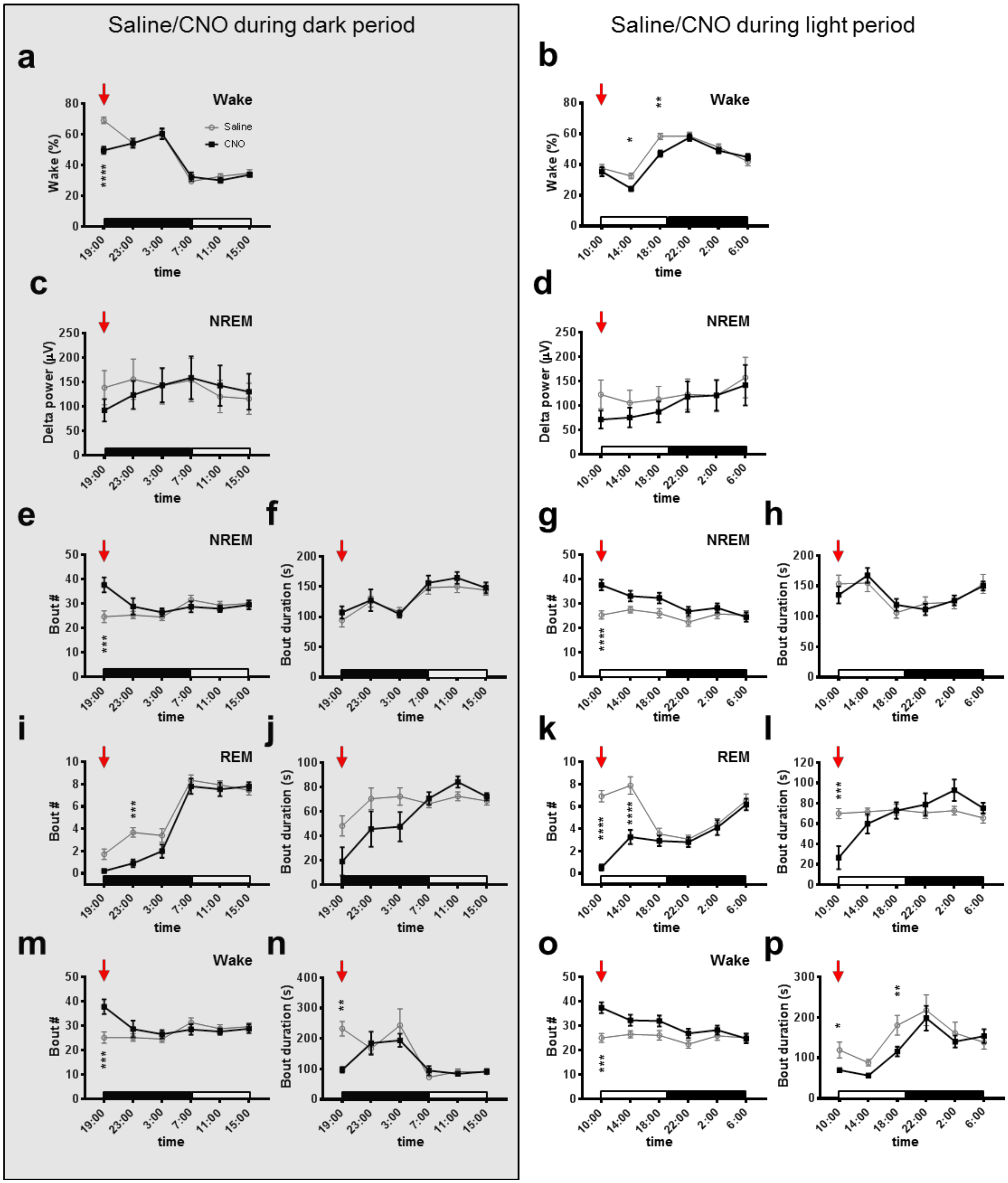

Supplementary Figure 5. Sleep-wake parameters after saline/CNO treatment in VLPO^{GAL}-hM3Dq mice. (**a,b**) Percent time spent in wake plotted at 4 h intervals after saline or CNO (0.3 mg/kg) treatment at 19:00 h (**a**) or 10:00 h (**b**); we used two-way RM ANOVA for the first 12 h after treatment for ‘time’ and ‘compound injected’, followed by Sidak’s *post hoc* test for graphs **a-p**. 19:00 h injections (**a**): time $F(2,32)=2.021$, $p=0.1492$, compound injected $F(1,16)=15.2$,

p=0.0013; n = 9 mice; 10:00 h injections (**b**): time *F*(2,48)=67.32, *p*<0.0001, compound injected *F*(1,24)=14.98, *p*=0.0007; n = 13 mice. (**c,d**) EEG delta power (0.5-4Hz) during NREM sleep after saline/CNO at 19:00 h (**c**) or 10:00 h (**d**); these were not statistically significant (n = 11 mice for 19:00 h injections; n = 13 mice for 10:00 h injections). (**e,g**) Number for NREM bouts after saline/CNO at 19:00 h (**e**) or 10:00 h (**g**); (19:00 h injections: time *F*(2,40)=4.772, *p*=0.0138, compound injected *F*(1,20)=6.068, *p*=0.0230; n = 11 mice; 10:00 h injections: time *F*(2,48)=1.184, *p*=0.3148, compound injected *F*(1,24)=14.52, *p*=0.0008; n = 13 mice). (**f,h**) Average duration for NREM sleep bouts after saline/CNO at 19:00 h (**f**) or 10:00 h (**h**); (19:00 h injections: time *F*(2,40)=5.338, *p*=0.0088, compound injected *F*(1,20)=0.1064, *p* = 0.7477; n = 11 mice; 10:00 h injections: time *F*(2,48)=18.48, *p*<0.0001, compound injected *F*(1,24)=0.02866, *p*=0.8670; n = 13 mice). (**i,k**) Number of REM sleep bouts after saline/CNO at 19:00 h (**i**) or 10:00 h (**k**); (19:00 h injections: time *F*(2,40)=11.58, *p*=0.0001, compound injected *F*(1,20)=15.49, *p*=0.0008; n = 11 mice; 10:00 h injections: time *F*(2,48)=12.2, *p*<0.0001, compound injected *F*(1,24)=54.39, *p*<0.0001; n = 13 mice). (**j,l**) Average durations for REM sleep bouts after saline/CNO at 19:00 h (**j**) or 10:00 h (**l**); (19:00 h injections: time *F*(2,40)=6.093, *p*=0.0049, compound injected *F*(1,20)=5.102, *p*=0.0352; n = 11 mice; 10:00 h injections: time *F*(2,48)=7.906, *p*=0.0011, compound injected *F*(1,24)=5.837, *P*=0.0237; n = 13 mice). (**m,o**) Number of wake bouts after saline/CNO at 19:00 h (**m**) or 10:00 h (**o**); (19:00 h injections: time *F*(2,40)=5.32, *p*=0.0089, compound injected *F*(1,20)=5.953, *p*=0.0241; n = 11 mice; 10:00 h injections: time *F*(2,48)=1.242, *p*=0.2979, compound injected *F*(1,24)=12.6, *p*=0.0016; n = 13 mice). (**n,p**) Average durations of Wake bouts after saline/CNO at 19:00 h (**n**) or 10:00 h (**p**); (19:00 h injections: time *F*(2,40)=1.64, *p*=0.2067, compound injected *F*(1,20)=5.237, *p*=0.0331; n = 11 mice; 10:00 h injections: time *F*(2,48)=19.39, *p*<0.0001, compound injected *F*(1,24)=12.29, *p*=0.0018; n = 13 mice). Red arrowheads indicate time of saline/CNO injection. All data are Mean ± SEM. *p < 0.5, **p < 0.01, ***p < 0.001, ****p < 0.00001.

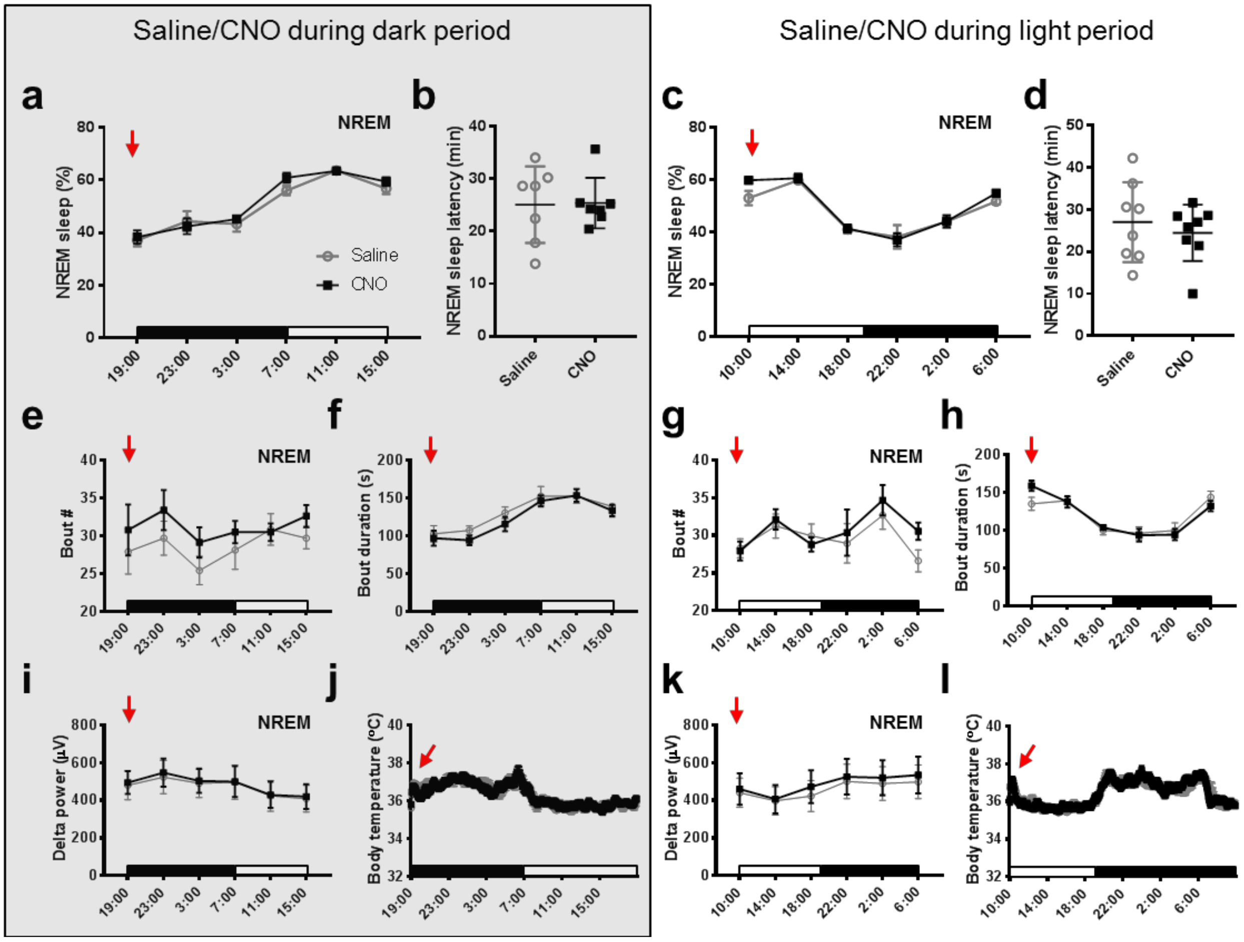

Supplementary Figure 6. NREM sleep parameters after saline/CNO treatment in VLPO^{GAL}-mCherry (control) mice. (**a,c**) Percent of time spent in NREM sleep at 4 h intervals for 24 h after saline or CNO (0.3 mg/kg) treatment at 19:00h (**a**) or 10:00h (**c**) and the respective NREM sleep latencies (**b,d**) in control VLPO^{GAL}-mCherry mice (i.e., they did not receive the hM3Dq DREADD), for comparison with Supplementary Figure. 4. Number (**e, g**) and mean durations (**f, h**) of NREM sleep bouts and EEG delta power (0.5-4Hz) in NREM sleep (**I, k**) in 4 h bins and body temperature (T_b) (**j,l**) in 5-min bins during the same 24 h periods. All data were analyzed using 2-way RM ANOVAs for 'time' and 'compound injected', except sleep latencies (**b,d**), which were analyzed using a Wilcoxon matched-pairs signed rank test (n = 7 mice for all conditions). There were no significant differences between saline and CNO treatment for any of the parameters. All data are presented as Mean ± SEM except sleep latencies which are Mean ± SD. Red arrowheads indicate time of saline/CNO injection.

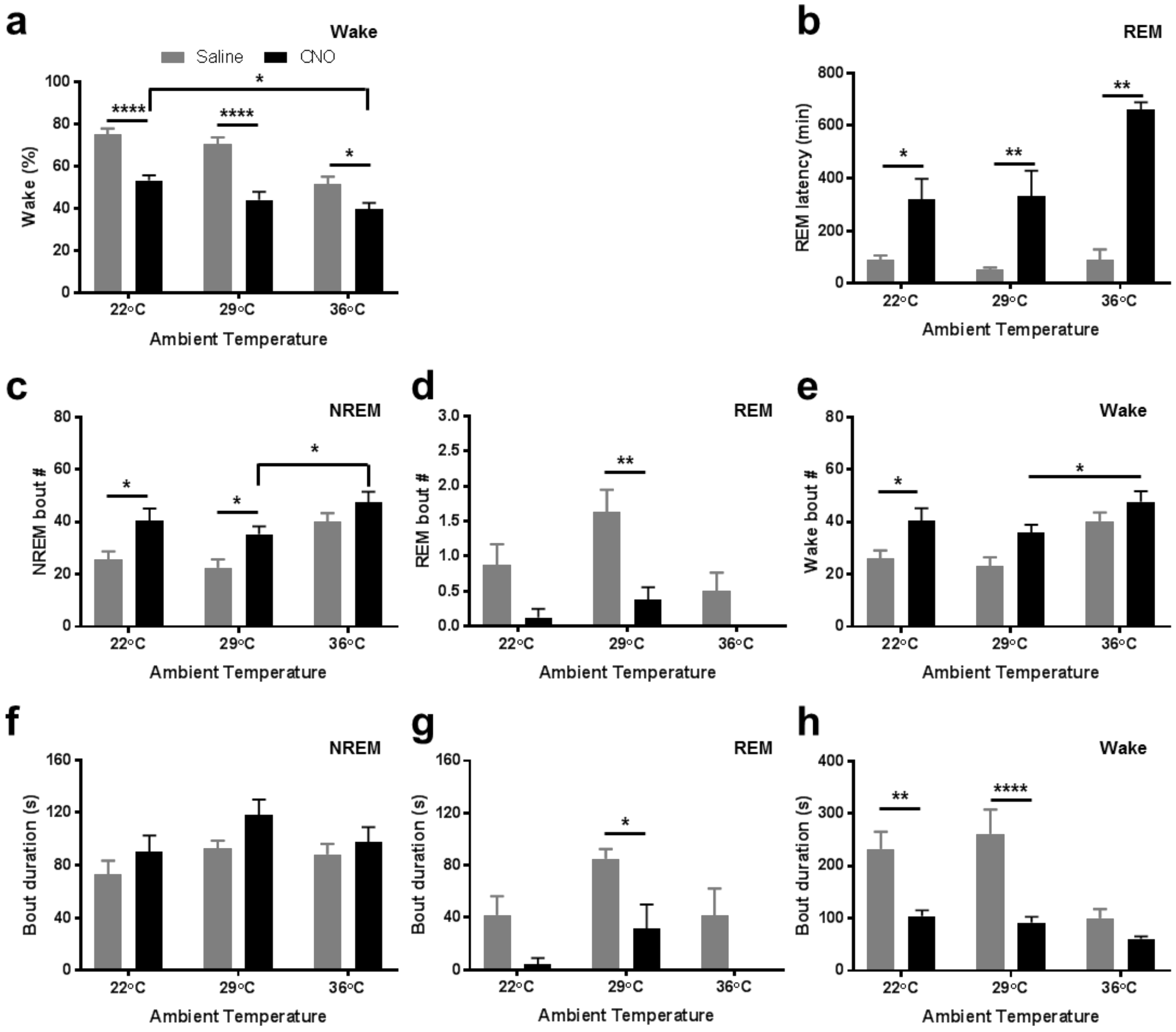

Supplementary Figure 7. Sleep parameters and body temperature after saline/CNO treatment in VLPO^{GAL}-hM3Dq mice at different ambient temperatures. (**a**) Percent of time spent in wake during the first 2 h in VLPO^{GAL}-hM3Dq mice (n = 8 mice) after saline or CNO (0.3 mg/kg) treatment at ambient temperatures 22ºC, 29ºC and 36ºC. Saline/CNO injections were performed at dark onset (19:00 h) and after the mice were exposed to the respective temperature for 45 mins; two-way RM ANOVA for 'ambient temperature' and 'compound injected', followed by Sidak's *post hoc* test (ambient temperature $F(2,28)=25.81$, $p<0.0001$, compound injected $F(1,14)=34.32$, $p<0.0001$; n = 8 mice). (**b**) REM sleep latencies in VLPO^{GAL}-hM3Dq mice (n = 8 mice) after saline/CNO treatment at ambient temperatures 22ºC, 29ºC and 36ºC; ordinary two-way ANOVA for the first 2 h after treatment for 'ambient temperature' and 'compound injected', followed by Sidak's *post hoc* test (ambient temperature $F(2,32)=2.553$,

p=0.0936, compound injected *F*(1,32)=32.99, *p*<0.0001; n = 8 mice). (**c-e**) Numbers for NREM sleep (**c**), REM sleep (**d**) and wake (**e**) bouts in $VLPO^{GAL}$-hM3Dq mice (n = 8 mice) after saline/ CNO treatment at ambient temperatures 22ºC, 29ºC and 36ºC; two-way RM ANOVA for the first 2 h after treatment for 'ambient temperature' and 'compound injected', followed by Sidak's *post hoc* test (NREM sleep: ambient temperature *F*(2,28)=11.16, *p*=0.0003, compound injected *F*(1,14)=11.53, *p*=0.0043; REM sleep: ambient temperature *F*(2,28)=6.877, *p*=0.0037, compound injected *F*(1,14)=14.58, *p*=0.0019; wakefulness: ambient temperature *F*(2,28)=10.46, *p*=0.0004, compound injected *F*(1,14)=11.36, *p*=0.0046; n = 8 mice). (**f-h**) Mean durations of NREM sleep (**f**), REM sleep (**g**) and wake (**h**) bouts in $VLPO^{GAL}$-hM3Dq mice (n = 8 mice) after saline/CNO treatment at ambient temperatures 22ºC, 29ºC and 36ºC different ambient temperatures; two-way RM ANOVA for the first 2 h after treatment for 'ambient temperature' and 'compound injected', followed by Sidak's *post hoc* test (NREM sleep: ambient temperature *F*(2,28)=4.484, *p*=0.0204, compound injected *F*(1,14)=2.458, *p*=0.1392; REM sleep: ambient temperature *F*(2,28)=4.821, *p*=0.0159, compound injected *F*(1,14)=17.25, *p*=0.0010; wakefulness: ambient temperature *F*(2,28)=13.1, *p*<0.0001, compound injected *F*(1,14)=17.93, *p*=0.0008; n = 8 mice). All data presented as Mean ± SEM. *p < 0.5, **p < 0.01, ***p < 0.001, ****p < 0.00001.

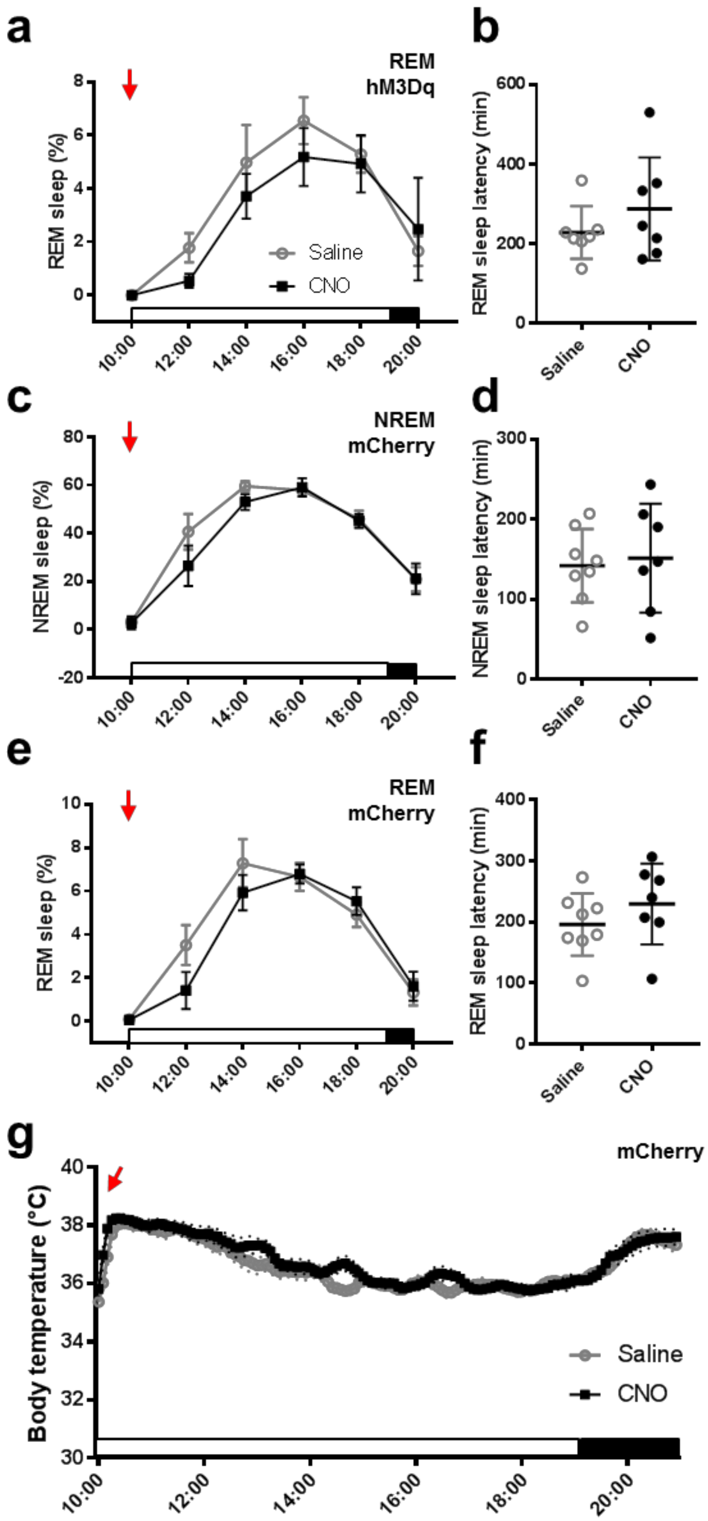

Supplementary Figure 8. Sleep and body temperature after saline/CNO treatment in VLPO^{GAL}-hM3Dq mice and VLPO^{GAL}-mCherry mice in a novel cage. (**a**) Percent of time spent in REM sleep every 2 h for 12 h after saline or CNO (0.3 mg/kg) treatment and cage change (at 10:00 h) in VLPO^{GAL}-hM3Dq mice (n = 7 mice) and corresponding REM sleep

latencies (**b**). Percent of time spent in NREM sleep (**c**) and REM sleep (**e**) every 2 h for 12 h after saline or CNO (0.3 mg/kg) treatment and cage change (at 10:00 h) in VLPO^{GAL}-mCherry mice (n = 7 mice) and corresponding NREM (**d**) and REM sleep latencies (**f**). Body temperature of VLPO^{GAL}-mCherry mice (n = 8 mice) every 5 min during the same 12 h period is presented in (**g**). Sleep amounts (**a,c,e**) and body temperature (**g**) were analyzed using a 2-way repeated measures ANOVA for 'time' and 'compound injected', while sleep latencies (**b,d,f**) were analyzed using a Wilcoxon matched-pairs signed rank test. There were no significant differences between saline and CNO treatment for any of the parameters. All data presented as Mean ± SEM, except sleep latencies which are Mean ± SD. Red arrowheads indicate time of saline/CNO injection and cage change.

		NREM		REM		Wake	
		mCherry	ChR2	mCherry	ChR2	mCherry	ChR2
1Hz photo stimulation	**% time**	36.0±4.7	61.1±2.5[c]	2.4±0.9	2.9±0.5	64.7±5.4	35.7±2.7[d]
(21:00-23:00h)	**Episode dur.**	371.7±40.5	398.4±36.5	101.3±14.6	76.3±13.7	704.5±159.8	234.8±27.0
n=10 ChR2; 6 mCherry	**Episode #**	7.3±1.1	11.8±1.0[a]	1.3±0.5	2.4±0.4	7.7±01.1	11.7±1.4[a]
	**FFT delta**	0.53±0.01	0.63±0.01[a]	0.26±0.03	0.43±0.01[b]	0.34±0.03	0.48±0.02[b]
	**FFT theta**	0.32±0.02	0.28±0.01[a]	0.43±0.02	0.38±0.01	0.31±0.01	0.26±0.01[a]

Supplementary Table 1. Sleep-wake behavior after optogenetic activation of VLPO^{GAL} neurons. Percent time, number and duration of bouts and EEG power spectra of individual sleep-wake stages during 1Hz photostimulation (21:00-23:00 h) in VLPO^{GAL}-mCherry mice (n = 6) and VLPO^{GAL}-ChR2 mice (n = 10). Data are Mean ± SEM. a = p<0.05, b = p<0.01, c = p<0.001, d = p<0.0001.

		% time			bout # (during 5 min)			bout duration (sec)		
		pre	during	post	pre	during	post	pre	during	post
NREM	sham inhib.	45.2±3.3	42.0±3.2	44.9±2.0	0.8±0.1	0.8±0.1	0.8±0.0	97.9±3.0	113.0±11.5	103.6±7.4
	laser inhib.	48.8±3.0	42.5±4.2	35.2±2.9 a,b,c	0.9±0.1	0.8±0.1	0.7±0.1	99.6±6.1	112.9±10.9	88.6±5.0
REM	sham inhib.	3.6±0.9	3.0±0.6	3.2±0.7	0.2±0.0	0.2±0.0	0.2±0.0	33.2±5.3	35.4±13.0	38.1±5.0
	laser inhib.	3.3±0.4	4.8±1.2	3.3±1.0	0.2±0.0	0.2±0.0	0.2±0.0	31.6±2.9	23.8±6.7	36.7±9.5
wake	sham inhib.	51.2±3.1	55.0±3.1	51.9±1.8	0.9±0.0	0.9±0.0	0.9±0.1	101.2±5.9	97.0±7.0	103.4±5.4
	laser inhib.	47.9±2.9	52.7±4.9	61.5±3.7 a,b,c	0.9±0.1	0.9±0.0	1.0±0.0	98.5±6.0	95.3±4.4	114.3±6.6 b

Supplementary Table 2. Sleep-wake behavior after optogenetic inhibition of VLPO^{GAL} neurons. Percent time, bout number and duration of individual sleep-wake stages 5 min before ('pre'), during ('during') and after ('post') optoinhibition or sham inhibition during the 12 h dark period in VLPO^{GAL}-ArchT mice (n=7). Laser inhibition or sham inhibition was applied for 5 min every 30 min with simultaneous EEG/EMG recordings. We used one-way RM ANOVAs to compare changes over time ('pre', 'during', 'post'), and Wilcoxon matched-pairs sign rank tests to compare treatment conditions ('sham', 'stim'). Data are Mean ± SEM. a = p<0.01 'pre' vs. 'post, b = p<0.05 'during' vs. 'post', c = p<0.05 'sham' vs. 'stim'.

Supplementary References

1 Franklin, K. B. J. & Paxinos, G. *The mouse brain in stereotaxic coordinates* Third Edition edn, (Elsevier, 2007).
